# Perinatal transmission of Lyme disease: A qualitative study investigating the research priorities of patients with Lyme disease in pregnancy

**DOI:** 10.1371/journal.pone.0294265

**Published:** 2024-02-06

**Authors:** Abeer Omar, Lindsay N. Grenier, Olivia Marquez, Sue Faber, Elizabeth K. Darling

**Affiliations:** 1 Trent/Fleming School of Nursing, Trent University, Peterborough, ON, Canada; 2 McMaster Midwifery Research Centre, McMaster University, Hamilton, ON, Canada; 3 LymeHope, Burlington, ON, Canada; 4 Department of Obstetrics and Gynecology, McMaster University, Hamilton, ON, Canada; University of Bologna / Romagna Local Health Authority, ITALY

## Abstract

**Introduction:**

Lyme disease is one of the most prevalent vector-borne disease in North America, yet its implications during pregnancy are poorly understood. Our knowledge of perinatal transmission of Lyme disease is limited due to the lack of robust epidemiological studies and longitudinal follow-up.

**Objectives:**

This study aimed to understand the research priorities of people who have experienced Lyme disease in pregnancy and the feasibility of recruiting this population for future studies on perinatal transmission of Lyme disease. We also sought to understand the barriers and enablers to participating in research on perinatal transmission of Lyme disease.

**Methods:**

We conducted a qualitative study using focus groups and interviews with people who had experienced Lyme disease during pregnancy. English speaking participants were recruited through an online survey. There was no geographic restriction on participation. The focus groups and the interview were recorded and transcribed. Data were analyzed using interpretive content analysis.

**Results:**

Twenty people participated in four semi-structured focus groups and one semi-structured individual interview. The majority of participants were from North America. Participants’ research priorities fell into five categories: transmission, testing, treatment, disease presentation, and education. All study participants expressed interest in future participation in research on Lyme disease in pregnancy and highlighted barriers and enablers to participation that could be addressed to facilitate future study recruitment.

**Conclusion:**

The research priorities identified in this research would be well addressed through prospective research. People who experience Lyme disease in pregnancy are invested in continued research into perinatal transmission of Lyme disease.

## Introduction

Lyme Disease (LD) is one of the most prevalent vector-borne diseases in North America [1, 2], and is found globally [1], typically in geographic clusters [3]. The geographic distribution and incidence rates have been increasing since LD’s identification in Connecticut in 1975 [4–7]. In high-risk regions like Maine, USA, and Ontario, Canada, annual incidence rates have been reported to be up to 130 and 85 per 100,000 people, respectively [4, 6]. Recent evidence in Canada indicates significant under-detection and under-reporting of LD cases across the country [8], and highlights the challenges of diagnosing this disease.

Lyme disease is caused by pathogenic bacteria from the Lyme *Borrelia burgdorferi sensu lato* species complex [9], transmitted through the bite of infected blacklegged ticks (*Ixodes scapularis*) [2]. The initial tick inoculation in humans may cause an erythema migrans (EM) lesion, also known as the bullseye rash, which can vary in size [10] and morphologic features [11]. However, the textbook bullseye rash is only present in 6–9% of cases and a homogenous red patch is present in 50–60% of cases [12–14], while 13–40% of LD cases do not correspond with any documented rash [15, 16]. Untreated LD can produce various symptoms, including fever, rash, fatigue, muscle and joint aches, facial paralysis, and arthritis. The infection may be associated with other non-specific symptoms [17], which further complicates an LD diagnosis [18], and up to 20% of infected individuals may have an asymptomatic or subclinical infection [19]. Diagnosis is based on clinical manifestations, the likelihood of exposure to black-legged ticks, and laboratory evidence of antibodies [20].

Diagnostic protocols vary across jurisdictions, but a recent review of European and American guidelines suggests a general consensus to recommend a standard two-tiered serology approach when EM is not present [21]. LD is easily treated with a 2–4 week course of oral antibiotics; however, if left untreated, it can have long-term impacts on joints, the heart, and the nervous system [2]. After successful treatment, some patients suffer from Post-Treatment Lyme Disease Syndrome (PTLDS), which includes symptoms like pain, fatigue, and brain fog, that persists for more than six months post-treatment [22]. Since this research is patient-focused, it is important to note that some research recommends abandoning the use of PTLDS to describe patients whose symptoms don’t improve after treatment protocols, in favour of “chronic Lyme disease,” “late Lyme disease,” or “late/chronic Lyme disease,” as PTLDS has a narrow definition that can exclude patients from receiving care or participating in research related to LD [23, 24].

### Lyme disease in pregnancy

The implications of LD in pregnancy are poorly understood. Pregnancy can increase vulnerability to vector-based infections due to a weakened immune system [25, 26], and infection in pregnancy can pose risks to the fetus [27, 28]. LD is a spirochetal infection, and other spirochetal infections, including syphilis and leptospirosis, are transmitted in utero and can have serious consequences for the fetus and newborn [29, 30]. Data on infection rates among pregnant people are sparse [1] and challenges with diagnosis coupled with poor understanding of LD in pregnancy may limit the detection of LD in people who are pregnant. There is limited evidence regarding vertical transmission of LD and the health outcomes of people with LD in pregnancy and their offspring [1, 31–33], and there are significant knowledge gaps relating to fetal infection and adverse birth outcomes [1]; however, the potential risk of maternal-fetal transmission of LD is publicly acknowledged by the US Centres for Disease Control (CDC), Health Canada, and the National Institutes of Health (NIH) [34–36]. A recent review of 31 studies investigating maternal-fetal transmission of LD found statistically probable transmission in 13 (42%) and possible transmission in 2 (6%) of the studies [37]. Adverse outcomes, including deaths, heart anomalies, and preterm birth, were lower when mothers had been treated with antimicrobials (74% with no antimicrobial treatment, 29% with oral antimicrobials, and 12% with intravenous antimicrobials) [37].

In humans, vertical transmission of Lyme disease to the fetus, neonate and/or placenta has been described in published papers, conference abstracts and medical textbooks. These include 19 cases of congenital infection in which Borrelia burgdorferi (Bb) or Borrelia species was identified in fetal or infant tissue upon autopsy (following miscarriage, stillbirth or neonatal death) utilizing various direct detection methods such as culture, PCR, microscopy and LD specific histological techniques [38–45]. An additional 15 cases of suspected or probable congenital Lyme infection and adverse outcomes have been reported [43, 46–55], including 2 cases in which Bb specific antibody was detected in the cerebral spinal fluid of symptomatic infants [48, 49]. Furthermore, 2 cases of live-birth in which Bb was identified by PCR in cord blood of infants whose mothers were treated [56], and 1 case of live-birth (twins) where one twin had IgG and IgM antibodies to Lyme disease in cord blood have been reported [57]. Bb has also been identified in placentas from both treated [43, 45, 50, 56–61] treatment not specified [62, 63], and untreated pregnancies [43]. One study identified Bb in placentas of 3 asymptomatic pregnancies whose Lyme two-tier serology was borderline (1) or negative (2), highlighting an important question of the possibility of silent maternal-fetal transmission of the spirochete [62].

Transmission via breastfeeding has not been documented [33, 64] However, Bb DNA has been identified by PCR in breastmilk from two lactating mothers who were untreated for Lyme disease in pregnancy [65]. Although transmission via breast milk has not been reported, it cannot be ruled out [66, 67]. There is currently minimal documentation of sexual transmission of LD [68] however data from animal models and human studies suggests that it may be possible, and further research is needed [69, 70].

Reported adverse pregnancy outcomes associated with gestational LD include placental infection, miscarriage, stillbirth, neonatal death, and intrauterine growth restriction. A heterogeneous range of newborn outcomes includes prematurity, respiratory distress, hyperbilirubinemia, hypotonia, sepsis and orthopedic, dermatologic, urologic, cardiac and ophthalmologic anomalies [31, 32, 39–55, 71, 72]. Treatment of LD in pregnancy was shown in one study to reduce the chances of adverse effects to 11% compared to >50% without treatment [1], suggesting that diagnosis and treatment of LD during pregnancy is important. Researchers have emphasized that patients with unrecognized or undiagnosed Lyme disease in pregnancy who are therefore not treated for LD should be those of greatest concern for clinicians [31].

Knowledge gaps regarding LD in pregnancy are due to the lack of robust epidemiological studies and longitudinal follow-up [1, 25]. To prepare for further research on perinatal transmission of LD, and in keeping with the importance of patient engagement in research priority setting acknowledged by national research funding bodies [73–75] we sought to understand the research priorities of people who have experienced LD in pregnancy. Our primary research question was: What are the research priorities of people who have had LD during pregnancy regarding LD in pregnancy? We were also interested in soliciting feedback to help us determine the feasibility of recruiting people with LD in pregnancy to examine the perinatal transmission of LD. Our secondary research question was: What would be the barriers and facilitators to recruiting people with LD in pregnancy to prospective research investigating the perinatal transmission of LD?

## Methods

This study reports the findings of the qualitative arm of a two-phase, mixed-method research project that examined the feasibility of conducting research on the perinatal transmission of LD. The initial quantitative phase of the study included an online survey to identify health outcomes associated with LD in pregnancy and to gauge potential interest in prospective research investigating the perinatal transmission of LD [76]. Survey participants were recruited through the websites and social media platforms of the McMaster Midwifery Research Centre and of LD-focussed organizations and health care providers in Canada and the United States. At the end of the online survey, participants who had had LD were invited to participate in online focus groups to share their perspectives on research about LD in pregnancy—the findings of this phase are the focus of this manuscript. Both quantitative and qualitative study phases were approved by the Hamilton Integrated Research Ethics Board (study #11222). Informed consent was obtained from all participants.

### Study participants

Inclusion criteria for focus group participants were a) a diagnosis or suspected diagnosis of LD and b) having been pregnant at least once, regardless of pregnancy outcome. We chose to include participants with suspected diagnoses given the documented challenges related to appropriate diagnosis of LD [77, 78].; however, only 9% of survey respondents with LD had a suspected diagnoses versus confirmed diagnosis (91%). There was no geographic restriction on participants’ place of residence. Individuals who participated in the online survey between September 25, 2020 and November 28, 2020 were recruited to participate in the focus groups.

### Data collection

Survey participants who met the eligibility criteria and indicated an interest in participating were emailed further study information and a consent form to be completed online prior to participation. Focus groups were scheduled when a minimum of 3 participants could attend, with a target size of 6–8 participants per group. Scheduling continued until all respondents had been scheduled or had been provided three reminders to participate. Focus groups were hosted online via Zoom between October 26, 2020 and December 16, 2020 and were digitally recorded.

The focus groups were facilitated by two trained and experienced researchers (AO, OM) using a semi-structured interview guide. The semi-structured interview guide had two primary questions: 1) What topics or areas of research do you consider priorities regarding LD in pregnancy? and 2) If you were pregnant, would you be willing to participate in a study involving collecting placental tissue, cord blood, or breast milk, and why or why not? Additional sub-questions were used to prompt elaboration on the enabling factors or barriers to facilitate future research. Demographic information was collected from focus group participants through an online survey administered using a secure web-based research platform, REDCap.

### Data analysis

The audio recordings of the focus groups were transcribed using Otter.ai, a web-based artificial intelligence transcription application. The transcripts were anonymous but potentially identifying information was not removed. We used interpretive content analysis [79, 80], using the conventional approach to analyze the transcripts of the focus groups [81]. We used an inductive approach to categorize the patterns and themes that we identified in the participants’ responses to the interview questions. This approach produces data-driven themes rather than fitting the data into researchers’ pre-conceptualized categories or theoretical analysis [80–82]. An experienced researcher (OM) analyzed the data, and the themes were then reviewed and refined by the principal investigator (EKD). An additional team member (LG), experienced in qualitative research, analyzed the transcripts, and independently identified themes to ensure rigor. The entire research team reviewed both sets of analyses and came to a consensus on the final themes and findings.

## Results

From our original online survey [76], 157 participants indicated that they were interested in participating in focus groups and were sent emails with information about the research project. A total of 20 people responded and participated in one of four focus groups or an individual interview. Focus groups were an average of 81 minutes in length (range 73–115 minutes), with 3–6 participants present. Due to dropout from scheduled focus group participants, we had one interview with a single participant, which lasted 22 minutes. The characteristics of the participants are shown in Table 1. The majority of participants had LD for more than ten years (80%), had at least two pregnancies (30%), had at least one child diagnosed with Lyme (55%), and were from Canada or the USA (80%).

**Table 1:** Demographic characteristics of focus group participant.

Participant Demographics	% (n)
	n = 20
Age	
20–29	10.0 (2)
30–39	20.0 (4)
40–49	30.0 (6)
50–59	40.0 (8)
Number of pregnancies	
1	15.0 (3)
2	30.0 (6)
3	25.0 (5)
4	25.0 (5)
5	5.0 (1)
Number of children diagnosed with Lyme	
0	35.3 (6)
1	23.5 (4)
2	29.4 (5)
3	11.8 (2)
Number of years participant has had Lyme	
< 1 year	5.0 (1)
1–2 years	0.0 (0)
3–5 years	5.0 (1)
6–10 years	10.0 (2)
> 10 years	80.0 (16)
Employment	
Employed, Part time	30.0 (6)
Employed, Full time	35.0 (7)
Not employed	35.0 (7)
Country	
Canada	40.0 (8)
USA	40.0 (8)
Australia	10.0 (2)
Ireland	10.0 (2)
Previous participation in Lyme research	
Yes	25.0 (5)
No	75.0 (15)

### Research priorities

The research priorities shared by participants fell into five broad themes: Transmission, Testing, Treatment, Disease presentation, and Education. These are described below and summarized in Table 2.

**Table 2:** Research priorities of people who have experienced Lyme disease and pregnancy.

Research areas	Specific priorities
**Transmission**	Investigate possible sexual, perinatal, and breast milk transmission
**Testing**	Improve accuracy and accessibility of testing; standardize testing during pregnancy
**Treatment**	Develop consistent treatment protocols for pregnant people and general population
**Disease presentation**	Ensure assessment of outcomes includes long-term follow-up; investigate associations between LD and pregnancy complications, and LD and adverse child health outcomes
**Education**	Improve training and education for healthcare practitioners about LD; support more interprofessional collaboration with alternative healthcare providers (i.e., naturopaths)

#### Transmission

Investigating the potential modes of transmission for LD was a large concern for participants. LD sufferers wanted clarity on whether LD is sexually transmissible:

*I know it’s not necessarily proven that it’s sexually transmitted at this point*. *But are there ways that we can make sure that it isn’t*? *Because you see whole families with it*? *And that’s the thing that catches my attention*, *right*? *It isn’t just the mother and the children*. *It’s the husband*, *the mother*, *and the children…so*, *I have to question the validity of someone saying to me that it isn’t necessarily sexually transmitted*. (Focus group [FG] 2)

Participants had similar concerns regarding disease transmission from mother to child through pregnancy, delivery, and breastfeeding since many participants with LD also had children who exhibited LD symptoms from a young age. The lack of clarity surrounding transmission led participants to question their decisions to breastfeed,

*You know*, *some of what I’ve read recently is that it can be transmitted through breast milk*. *And*, *you know*, *I thought I was giving my kids the best*, *the best chance by*, *you know*, *nursing all three of them*. *And now I’m going okay*, *well*, *if I hadn’t done that*, *would they still be all*, *you know*, *different*? (FG 3)

#### Testing

Testing for LD was also identified as a priority area of research which included improving the accuracy of testing. Many participants shared skepticism towards the accuracy of testing methods, as they had received mixed results from different testing methods. Improving the accuracy of testing would help improve disease diagnosis, which participants saw to be key in appropriately treating and potentially limiting perinatal transmission of LD. Participants also noted that a timelier diagnosis of LD means faster treatment and, ideally, fewer health impacts for the patient. Participants identified a need for research to clarify diagnostic criteria for the disease other than laboratory results. Research focussed on methods for collecting samples and screening for LD specifically in pregnant people was a priority. One suggestion was to test the newborn for LD using the placenta. One participant suggested that testing for LD should be included in the standard testing conducted during pregnancy. As they expressed, "*I got screened for syphilis*, *I got screened for HIV*, *why are we not screening every pregnant [person] in this world for Lyme disease*?” (FG 2) Participants suggested that future research also needs to address the accessibility of screening for LD generally, as well as for expectant people due to their vulnerable status.

#### Treatment

Clear treatment protocols need to be developed since there are currently inconsistencies in participants’ experiences with the treatment of LD. One participant stated,

*So*, *coming to some sort of consensus*. *We need to figure out what the proper treatment is*. *Because right now we’ve got everything from Lyme disease doesn’t even exist*, *or it doesn’t exist here*, *to you need to take one pill*, *all the way to you need to take years of antibiotics*. *So*, *it’s quite a large spectrum*. (FG 6)

Participants also wanted a standard treatment protocol for the prevention of transmission during pregnancy: *“Yeah*, *for instance*, *you know*, *like*, *getting treated for herpes*. *You know*, *prior to giving birth*, *I would have done the same for Lyme if I known I had had it*, *you know*.*” (FG 2)*. Participants were concerned that LD treatments during pregnancy should be effective, yet safe for the mother and fetus, minimizing the risk of negative side effects.

Disease presentation, both in pregnancy and in children, was a major topic of interest for participants. Participants shared their own anecdotal experiences of comorbidities that they viewed as potentially associated with LD and identified a need for higher-quality evidence regarding whether LD increases the risk of pregnancy complications, such as preeclampsia and miscarriage. Participants were interested in knowing if LD could cause other conditions, even after LD treatment, and how it would present if transmitted perinatally. Participants emphasized that identifying the presence of LD acquired at birth may require a long follow-up period after birth. As one participant explained,

*I mean*, *in a lot of research that I have read*, *it’s focusing mostly on symptoms…after birth*. *Not all symptoms show up right after birth*. *And like I said*, *they start sometimes after two years old*. *So*, *they need to be followed longer than just right after birth*. *(FG 3*)

#### Education

Finally, participants were adamant that health professionals need better education and training on LD to increase the successful diagnosis and treatment of LD. As one said,

*[L]ooking back*, *one of the hardest things is that I [obviously had LD]*, *but my OB did not pick up on it*. *And if she were Lyme literate she absolutely would have*. *And then I could have been treated during pregnancy*. *So*, *I do think one of the biggest things is that OBs in general have no idea that this even occurs*. *(FG 3*)

Participants also proposed that collaboration among health professionals would be beneficial. One participant pointed out that naturopaths are at the forefront of treating and diagnosing Lyme, but naturopaths and other alternative care providers are typically outside the medical system. Therefore, improving collaboration between a variety of healthcare professionals might be a way to improve LD diagnosis, treatment, and support.

### Barriers & enablers to research participation

Discussion of barriers and enablers to research participation led to input from participants regarding general interest levels in research on LD in pregnancy and suggestions for recruitment strategies in addition to the identification of barriers and enablers of participation. These four topics are discussed below. The barriers and enablers are also presented in Table 3.

**Table 3:** Barriers and enablers to research participation for people with Lyme disease in pregnancy.

Barriers	Enablers
• Concerns about privacy and data/information security • Accessibility/capacity to participate • Institutional barriers • Collection of biological samples • Health risks	• Institutional buy-in • Anonymity • Incentives & costs • Study Methods

All study participants indicated that if they were eligible, they would participate in future research studies recruiting pregnant individuals with LD. This included the willingness to participate in the collection of biological samples, including the placenta/placental tissue, cord blood, and breast milk. Participants cited two main motivations: altruism and the potential to gain information about their child. Participants’ desire to participate in future research arose from believing that it would be helpful to all to learn more about the disease, particularly because the research would have the potential to help people diagnosed with LD in future. As one participant explained, “*I think a lot of us are wanting to not only help ourselves but help others because we live in these shoes every day*.” (FG 2) Many participants had suffered or watched loved ones suffer from LD and wanted to help others in similar situations. Secondly, participation in research was seen as a potential opportunity for parents to gain information about their child’s LD status or diagnosis earlier.

Participants identified five factors that would impede their participation in future LD research (see Table 3). Participants were wary about how their data and information would be used and would not participate if their data were to be shared with any third parties. Accessibility and capacity to participate in the study program was another potential barrier, as long distances, lack of transportation, and the fatigue often experienced by those with LD and pregnancy could pose challenges to those wanting to participate. Institutional factors, like the cooperation of healthcare providers, hospitals, and clinics to support the research, could potentially hinder participation. Unsupportive healthcare providers and the stigma that can be associated with LD in the medical community could be an impediment, particularly if the support of medical professionals was required to collect the biological samples. Some participants had previously attempted to collect their placenta and other samples for a different research study and were unable to receive the biological materials. Finally, participants expressed reticence to collect samples during pregnancy if this collection would potentially increase the risk of complications such as miscarriage.

Participants also identified several factors that would facilitate their participation in future LD studies: institutional buy-in, anonymity, study methods, and incentives and costs (Table 3). Having institutional buy in from hospitals, clinics, and medical professionals, as well as from designated labs and locations for any other study procedures would make participants feel more comfortable and less concerned about potential stigmatization. Additionally, participants would appreciate the ability to participate anonymously and not have to share any diagnoses with health professionals outside the study. The study methods could make a great difference in enabling people to participate in research. Keeping the requirements of participants simple, realistic, and not onerous will enable participation. Additionally, participants were critical of certain laboratory tests used to detect for LD, so they would recommend not using an ELISA test. Participants wanted to know that the research would contribute meaningfully to the understanding, diagnosis, and treatment of LD, and be widely disseminated to help others. Finally, financial incentives and/or reducing participation costs would be another factor to facilitate greater participant recruitment. A suggested non-financial incentive would be for there to be long-term medical follow-up for participants (including babies born to participants with LD), so that if a positive diagnosis is discovered during the study, participants have an easy route to access medical care.

Finally, participants recommended several recruitment strategies for future LD research from their perspective as research participants in our current study. Using targeted advertising and social media sites like Facebook were highly endorsed and perceived as having wide reach. Participants suggested that contact with LD patients could be facilitated through consent to contact lists maintained by hospitals and other healthcare institutions. Lyme literate medical doctors (LLMDs), fertility specialists, naturopaths treating and caring for Lyme patients, and midwifery practice groups were also identified as reliable routes for recruitment, since they can directly recommend and advertise the study to patients/clients. Organizations supporting LD patients and LD research would also be potential routes for recruitment through email lists or advertisements posted on websites or social media.

## Discussion

We found that people who have experienced LD in pregnancy are in strong favor of research being done in this area and are interested in participating in future research. Participants identified a variety of research questions that would be important to address, and these fell within five priority areas: transmission, testing, treatment, disease presentation, and education of healthcare practitioners. Participants additionally described factors which would enhance or hinder their participation in future research and made suggestions for recruitment strategies.

The patient research priorities identified by our participants align with research gaps identified by a systematic review on LD in pregnancy [1]. Similar gaps included testing accuracy and diagnostic protocols, evidence for vertical transmission of LD, the impact of LD on the fetus, and potential associations between LD and adverse birth outcomes [1, 83, 84]. Our findings also align with previous research examining the experiences of parents of children with LD, which identified that improved diagnostic testing and treatment guidelines are necessary to improve the experiences of families living with LD [85]. Other literature has identified the need for safe and effective treatment, causes of symptoms post-treatment, better-informed healthcare providers, and more public awareness [84]. That patient priorities identified by our research align so well with known gaps in the literature demonstrates that patients’ experiences have made them well aware of knowledge gaps.

Governmental agencies have been slow to engage patients in setting priorities for LD research, though this appears to be starting to shift. The Federal Framework on Lyme Disease was released by the Government of Canada in May 2017, which resulted in the Canadian Lyme Disease Research Network and sought to include patients, family representatives, and informal care providers [86]. In 2019, the National Institutes of Health (NIH) Tickborne Diseases Strategy Planning Team published the NIH Strategic Plan for Tickborne Disease Research in the USA [87]. In 2018, the National Institute for Health Care Excellence (NICE) in the U.K. published Guidelines on LD, which included research recommendations [88]. These governmental agencies commonly seek to address gaps in the knowledge base of LD, improve testing, diagnostic accuracy, prevention, and treatment at all disease stages, and learn more about modes of transmission [86–88]. The degree of patient involvement in the creation of these research priorities and recommendations is unclear.

In 2020, the U.S. Tick-Borne Disease Working Group (TBDWG) recommended: ‘Further evaluation of non-tick bite transmission of Lyme disease, for example, maternal-fetal transmission.’ The report also stated, ‘Similarly, additional studies of potential congenital Lyme disease, and of persistent Lyme disease in undiagnosed and untreated infants resulting from maternal transmission of B. burgdorferi, could be helpful, as could patient registries’ [89]. A subsequent 2022 Clinical Presentation and Pathogenesis subcommittee report to the TBDWG has addressed pregnancy and LD in much more detail and subsequently recommended establishment of interim guidelines for evaluation, testing, and management of infants born to mothers with LD diagnosed during their pregnancy, funding opportunities for prospective cohort studies of people infected with LD during pregnancy and their offspring, and funding to support repositories of biological samples from pregnant and lactating people, and their offspring [90].

Non-governmental organizations appear to be more patient-centred in their research and recommendations regarding LD. For example, in the U.K. the James Lind Alliance entered into a priority setting partnership with the charity, Lyme Disease Action, to develop a top 10 priorities/questions for LD research to address based on a 2011 survey of patients and clinicians and a review of the literature [91, 92]. MyLymeData, a patient-powered and -centered LD research community in the U.S. created by Lymedisease.org [93], surveyed patients to seek feedback on the NIH strategic priorities pertaining to LD and found major discrepancies and disagreements between the priorities of the NIH and those of patient respondents [94]. Major recommendations from this research relevant to our findings indicated the NIH should increase funding for LD research, there should be better patient representation on grant-funding panels, and research should be conducted in partnership with LD patients [23]. In 2015, MyLymeData launched the only national registry and research platform for LD patients, which had enrolled 15,000 patients as of 2019 [93]. The research project was developed and run by patients, and assumes that patients are the best experts in their own illness [93]. Engaging patients with expert knowledge and lived experiences dealing with LD in the healthcare system provides unique perspectives that strengthens research quality [95]. Identifying and focusing on the priorities of patients also follows the Canadian Institutes of Health Research’s Strategy for Patient-Oriented Research, which ultimately can improve health care practice, policies, and health outcomes [95].

### Strengths & limitations

A strength of our study was the open, worldwide recruitment. However, the findings may be more representative of American and Canadian priorities due to the majority of participants being from these countries. Our findings may be less applicable to other jurisdictions, as peoples’ experiences and perceptions are shaped by the health system where they live and seek care. Most of our participants had been diagnosed with LD more than ten years ago and we did not collect data in this phase of the study to describe the timing of LD diagnosis in relationship to the timing of pregnancy or LD diagnosis in children. The demographics of our participants suggest that they likely were not representative of all people who have LD during pregnancy. Our participants may have experienced greater morbidity from LD and higher rates of LD in their offspring than average, which likely influenced our findings. However, the perspectives of people with chronic LD are particularly valuable in identifying the limits of current medical understanding about this disease. The online focus group format may have prohibited the participation of rural dwellers and people with low socioeconomic status. Our findings are also potentially limited by selection bias, as those who had negative experiences may have been more likely to participate as a way to share their experience. Previous research has shown that people with LD often describe being treated in ways that were condescending, dismissive, or patronizing [96]. A strength of our study was that participants expressed appreciation of the opportunity to share their concerns and priorities, which enabled us to give voice to a group that has often not been listened to. However, it is possible we didn’t hear from individuals who have lost trust in healthcare and research due to their negative experiences with the healthcare system. We chose to take an inclusive approach to eligibility to participation so enrollment in this study didn’t require proof of diagnosis. Given the challenges of LD diagnosis, this may be considered a strength, but the research priorities identified may have differed if we had implemented more rigid eligibility criteria.

### Implications for research

The priorities identified by participants in our study should provide direction for future LD research. To enhance the recruitment of people with LD in pregnancy in research, researchers can attend to the barriers and enablers identified (Table 3). Researchers should design their studies to reduce barriers to participation, for example, by maximizing institutional support, ensuring methods that are not onerous for participants, providing incentives and limiting costs to participate, safeguarding patient data and anonymity, and minimizing health risks during pregnancy. It appears that conducting prospective research with those with LD in pregnancy is feasible based on their willingness to participate in future research.

Research focusing on improving both treatment within the medical system and collaboration with alternative practitioners may be another potential area of research. Echoing our participants’ experiences, the literature demonstrates that physicians are not well educated about LD. Prior research found that clinicians demonstrated basic knowledge of LD, had suboptimal practices, misinterpreted Western blot tests [97, 98], and struggled with diagnosing or conceptualising chronic LD [99]. Furthermore, our participants desired clinicians to be more collaborative with alternative healthcare providers. Many participants were seeking care primarily from naturopaths, as their needs were not getting met in the dominant medical system. Research shows that LD patients sought alternative methods when diagnostic procedures and treatments didn’t resolve their symptoms [100]. Their escalating health issues, and the stigma they faced led to feelings of desperation and abandonment, ultimately leading them to seek alternative practitioners outside the medical system [100]. There is potential for furthering knowledge if researchers and research funders ensure that approaches to care that are being used by complimentary and alternative medicine have the same opportunities for research evaluation as allopathic treatments.

### Conclusion

People who experience LD in pregnancy identify a broad range of research gaps that they believe are important to address. Priority topics span Transmission, Testing, Treatment, Disease presentation, and Education. Our findings indicate that some of these priorities would be well addressed through prospective research studies and that this patient population would be willing to participate in such research.

## List of abbreviations

LD: Lyme disease
EM: erythema migrans
PTLDS: Post-treatment Lyme disease syndrome
CDC: Centres for Disease Control
NIH: National Institutes of Health
FG: focus group
LLMDs: Lyme literate medical doctors
NICE: National Institute for Health Care Excellence
TBDWG: Tick-borne disease working group
